# A functional Magnetic Resonance Imaging study of patients with Polar Type II/III complex shoulder instability

**DOI:** 10.1038/s41598-019-42754-1

**Published:** 2019-04-18

**Authors:** Anthony Howard, Joanne L. Powell, Jo Gibson, David Hawkes, Graham J. Kemp, Simon P. Frostick

**Affiliations:** 1 Trauma & Orthopaedic Surgery, School of Medicine, University of Leeds, Leeds, UK; 2 Department of Psychology, Edge Hill University, Ormskirk, UK; 3 Physiotherapy Department, Royal Liverpool University Hospital, Liverpool, UK; 4 Department of Molecular and Clinical Cancer Medicine, University of Liverpool, Liverpool, UK; 5 Department of Musculoskeletal Biology and Liverpool Magnetic Resonance Imaging Centre (LiMRIC), University of Liverpool, Liverpool, UK

**Keywords:** Decision, Translational research

## Abstract

The pathophysiology of Stanmore Classification Polar type II/III shoulder instability is not well understood. Functional Magnetic Resonance Imaging was used to measure brain activity in response to forward flexion and abduction in 16 patients with Polar Type II/III shoulder instability and 16 age-matched controls. When a cluster level correction was applied patients showed significantly greater brain activity than controls in primary motor cortex (BA4), supramarginal gyrus (BA40), inferior frontal gyrus (BA44), precentral gyrus (BA6) and middle frontal gyrus (BA6): the latter region is considered premotor cortex. Using voxel level correction within these five regions a unique activation was found in the primary motor cortex (BA4) at MNI coordinates -38 -26 56. Activation was greater in controls compared to patients in the parahippocampal gyrus (BA27) and perirhinal cortex (BA36). These findings show, for the first time, neural differences in patients with complex shoulder instability, and suggest that patients are in some sense working harder or differently to maintain shoulder stability, with brain activity similar to early stage motor sequence learning. It will help to understand the condition, design better therapies and improve treatment of this group; avoiding the common clinical misconception that their recurrent shoulder dislocations are a form of attention-seeking.

## Introduction

Approximately 2% of the population have instability of the shoulder joint^1–3^. Shoulder instability is an inability to maintain the humeral head in the glenoid fossa, associated with discomfort, slipping or a sense that the shoulder is unstable and dislocatable^4^. The aetiology of shoulder instability is complex. There are three interrelated causes: muscle patterning dysfunction, structural defects that arise from trauma, and structural defects acquired through atraumatic processes^2^. Traumatic dislocations in young patients form the largest group^5,6^. However, in the authors’ experience a significant number of patients develop a complex instability that is often resistant to treatment.

Complex shoulder instability, as a condition, has rarely been included in shoulder instability classifications^7^, whose focus is usually on the traumatic aetiology^2,8–10^. However, shoulder instability is a dynamic process and patients who have abnormal muscle patterning may subsequently develop structural pathology. This is the basis of the Stanmore classification which acknowledges that these multi-factorial causes form a continuum. The classification defines three groups within a triangle: Polar Type I (traumatic structural), Polar Type II (atraumatic structural), and Polar Type III (muscle patterning non-structural)^2^. Positioning patients within the triangle supports a more objective description, and provides a representation of the interrelationship of causal factors.

Patients with complex shoulder instability are often mis- or under-diagnosed, with the condition often being seen as self-induced^2,11–13^. The treatment of shoulder instability depends upon an accurate assessment of each case, and the selected treatment modalities must reflect the contributing factors. In patients with complex shoulder instability, addressing the abnormal muscle patterning through physiotherapy, with the aim of strengthening the rotator cuff and the scapular musculature^14^, is the first line treatment^15^. However 30–40% of patients will not respond, and a large cohort of these tend to be female and 17–25 years of age, a group in which the instability has a marked effect on quality of life^16,17^. They are often then labelled as attention-seeking, or else receive inappropriate surgery which also fails to resolve their symptoms^15,18^. Physiotherapy, and other treatment strategies that incorporate visual feedback about motor performance^19^, have had success in the treatment of complex shoulder instability. This leads us to hypothesise that central cortical activation contributes to the instability. Indeed, there is evidence of muscle compensatory strategies in other shoulder conditions, such as rotator cuff tears^20^.

Shoulder movement relies on limb proprioception, the brain’s knowledge of the location of the upper limb in time and space independent of vision^21^. Proprioception is required for upper limb motor control^22^, especially involving small or precise co-ordinated movements^23^, and plays an important role in joint stability; importantly a reduction in proprioception has been linked to shoulder instability^24^.

The primary motor cortex has been studied for many years^25^. It was first thought that motor control was manifest in well-ordered discrete cortical areas, as conveyed by the iconic motor homunculus^26^. However, there has been a paradigm shift towards an understanding that cortical organisation is more complex^27,28^, particularly with regard to maintaining joint stability. The non-invasive technique of functional magnetic resonance imaging (fMRI) has been important in developing this new understanding, and has also led to clinical important developments in conditions such as Alzheimer’s and Parkinson’s disease^29^. fMRI studies of motor function in stroke^30–32^, amputees^33–35^, and movement dystonia^36^ have revealed adaptive changes with bilateral activation and cortical re-organisation in the sensorimotor areas, the supplementary motor areas and the cerebellum^37^.

Little fMRI work has been done on shoulder movement and its disorders. One study found decreased brain activity in the motor network and different areas of activations in patients with recurrent anterior shoulder instability^38^. Two studies on shoulder apprehension have demonstrated structural changes in the sensorimotor areas using a visual task to evoke shoulder apprehension^39,40^, and abnormalities in task-correlated functional connectivity, measured in the resting state, related to viewing images of shoulder movement^39,40^. No studies have considered patients with complex shoulder instability. Given the increasingly recognised importance of neural reorganisation in other conditions, we set out to explore the neural correlates of motor control in patients with complex shoulder instability using fMRI.

## Methods

### Participants

**Figure 1:**
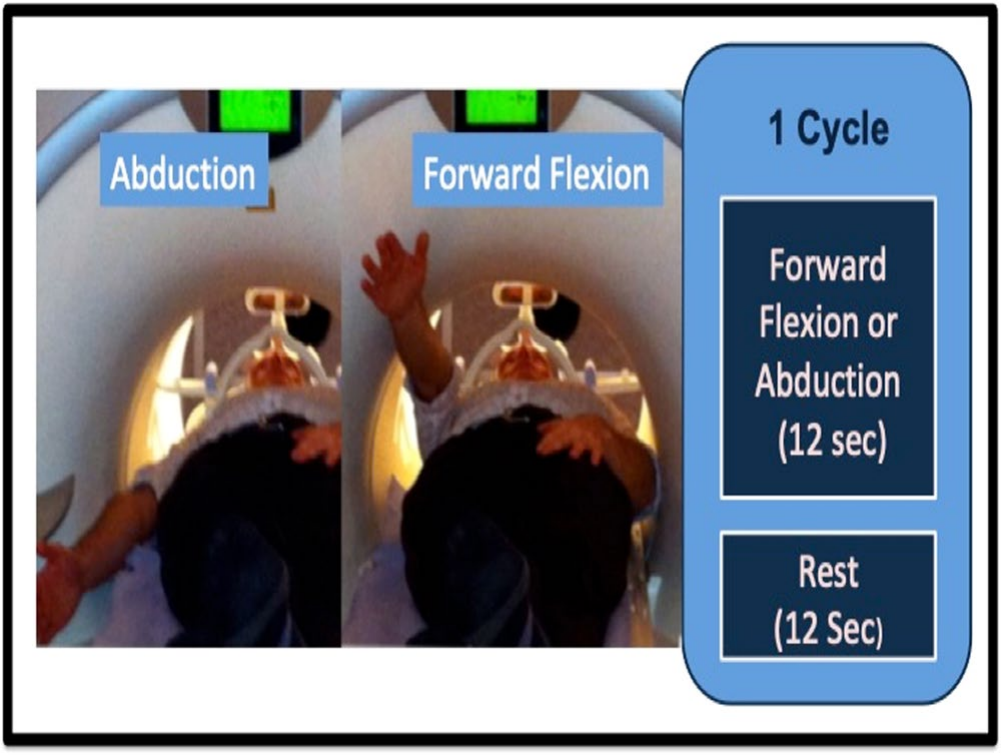
Movements of forward flexion and abduction in the scanner [14].

Patients were recruited with Polar Type II/III shoulder instability, confirmed by the senior surgeon (SPF) and physiotherapist (JG). The diagnosis was made on the basis of patient history, clinical examination, imaging (radiograph/MRI) and arthroscopy [8]. In total, 16 Polar type II/III patients were recruited along with 16 controls with no history of shoulder pathology, age-matched (as age can influence cortical representation)^41^; 4 individuals in each group wrote with their left hand [9]. Twelve of the sixteen patients experienced Polar Type II/III in the right shoulder; the remaining four who experienced Polar Type II/III in the left shoulder were also the group who wrote with their left hand. The patient group represented almost all of the eligible patients treated at our specialist centre over a four-year period. The sample size is consistent with that used in previous studies to yield sufficient statistical power^30,42–44^. Exclusion criteria included collagen disorders such as Ehlers-Danlos Syndrome; previous significant surgery; previous trauma; MRI exclusion factors (e.g. cardiac pacemaker), neuromuscular conditions, multiple sclerosis and any other possibly confounding brain pathology. No participant had been treated with psychoactive medication, which may have a confounding influence^45^. St Helens & Knowsley Teaching Hospital NHS Trust Local Research Ethics Committee granted ethical approval for study. All participants gave signed informed consent, and our work was performed in accordance with the relevant guidelines and regulations. Consent for images to be used in an online open-access publication was obtained, Fig. 1.

### Behavioural measures

The Oxford Shoulder Instability Score (OSIS)^46–48^ and the Western Ontario Shoulder Instability Index (WOSI)^46,49^, were chosen to evaluate the participants’ functional status: WOSI scores range from 0 to 2100 with 0 representing a normal score; OSIS scores range from 12–60 with 12 representing normal. For inclusion in the control group participants needed to present with a score of 0 on WOSI and 12 on OSIS.

### Experimental paradigm

The movement protocol contrasted forward flexion and abduction against rest, Fig. 1. A mixed block/event-related fMRI design was used. Overall there were 20 blocks of movement. Each block was 12 seconds, a duration comparable with other mixed design studies^50–52^. In a block participants undertook either forward flexion, abduction or rest; controls moved the right, patients moved the arm subject to spontaneous shoulder dislocation. The movement sequence was randomised to reduce the confounding effect of learned behaviour^53^. Subjects were shown the pathway and speed of movement (2 Hz) and how to lock their elbow and wrist, in order to reduce inter-subject variability in movement strategy. Close observation was made for head movement, and if detected the paradigm was restarted. The paradigm was repeated three times, only the third attempt data being used for the analysis. The required movement (i.e. forward flexion, abduction or rest) was communicated by projecting coloured lights onto a screen visible from inside the scanner using Presentation software (NeuroBehavioural Systems, California).

### Data acquisition

Images were acquired using a Siemens Trio 1.5 T whole-body MR system, with an 8-channel head coil. Functional images were obtained using a T_2_-weighted gradient echo EPI sequence (TE = 35 ms, TR = 3000 ms, flip angle = 90°, slice thickness = 3 mm, 0.3 mm gap, matrix = 64 × 64, FOV = 192 mm, in-plane resolution 3 × 3 mm). Forty-three axial slices were acquired parallel to the AC–PC line covering the whole brain. Additional high resolution T_1_-weighted anatomical images were acquired sagittally (TE = 5.57 ms, TR = 2040 ms, flip angle 8°, FOV = 256, 176 slices, voxel size 1 × 1 × 1 mm). Head restraints were used to control head movement.

### Image pre-processing and analysis

Statistical Parametric Mapping Software package (SPM 12) (University College London)^54–59^ was employed for realignment, normalisation, smoothing and to create statistical parametric maps of significant regional BOLD response based on the statistical analysis. The image time series were realigned after discarding the first two images (acquired before the MR signal reached steady state). As the patient’s affected side was used for the movement protocol, functional images for the few left-sided (and left-handed) subjects were flipped prior to processing. This was to ensure cortical activation contralateral to the affected side was matched across all individuals. For each subject a mean functional image volume was created from the realigned image following sinc-interpolation transformation. T_1_-weighted images were co-registered to the mean functional images then segmented. The grey matter segment was then normalised to the template provided by the Montreal Neurological Institute (MNI) within SPM12. The resultant parameters were used to transform the T_1_-weighted and functional images into MNI space. Prior to statistical analysis, the normalised images were smoothed with an isotropic 6 FWHM Gaussian kernel. A symmetrical version of the MNI template within SPM12 was created by averaging the flipped and un-flipped images using the Masking toolbox (http://www0.cs.ucl.ac.uk/staff/g.ridgway/masking/); this was used during the ‘normalised’ and ‘segment’ processing. Two contrasts were computed at the first level; forward flexion >rest and abduction >rest. Movement parameters of the head were entered as covariates in the model along with a grey matter mask. Individual contrast images were imported into a second level analysis.

Technical development scans (N = 5) to test the movement protocol demonstrated that a good range of movement (forward 40 degrees and abduction 20 degrees) was possible within the confines of the MRI scanner with acceptable movement artefacts. The protocol produced activation in Brodmann area (BA) 5, involved in somatosensory processing, consistent with other upper limb movement studies^60^, along with other areas involved in movement including primary motor cortex (BA4), premotor cortex (BA6), somatosensory association cortex (BA7); and dorsolateral prefrontal cortex [DLPC] (BA9 and BA46)^61^. Reproducibility was demonstrated by rescanning a control 13 months following the initial study (*P* < 0.001, FWE, t values 5.71–10.21)^62^.

In the final analysis a full-factorial model was used to identify areas of activation for forward flexion and abduction across the whole group and to test for differences in activation between control and patient groups. An F-test was used to test for differences in activation between forward flexion and abduction (FWE, *P* < 0.05). Separate t-tests were used to identify areas of greater activation for movement versus rest in patients and controls and in controls versus patients. The statistical parametric maps were interpreted after applying a family-wise error (FWE) correction with *P* < 0.05 (cluster size of K ≥ 10) using the toolbox bspmview (http://www.bobspunt.com/bspmview/) in SPM12. Regions were identified using SPM Anatomy Toolbox (Version 2.1)^63–67^ and WFU PickAtlas^68,69^. A total of five regions were identified from this model, from which masks were created using bspmview. Voxel level correction (FWE, *P* < 0.05) was applied using the five masks as ROIs for the contrast patients >controls for all movements versus rest.

## Results

The mean age of the patient group was 24.2 ± 6.0 years (15 female/1 male), and of the controls 23.8 ± 5.1 years (15 female/1 male)^18^. In both groups 4 individuals wrote with their left hand. In the patient group, the mean OSIS score was 17.5 ± 13.1 (range: 0–48) and the mean WOSI score was 1164 ± 558 (range: 74–2100). One patient had normal WOSI and OSIS scores.

**Figure 2:**
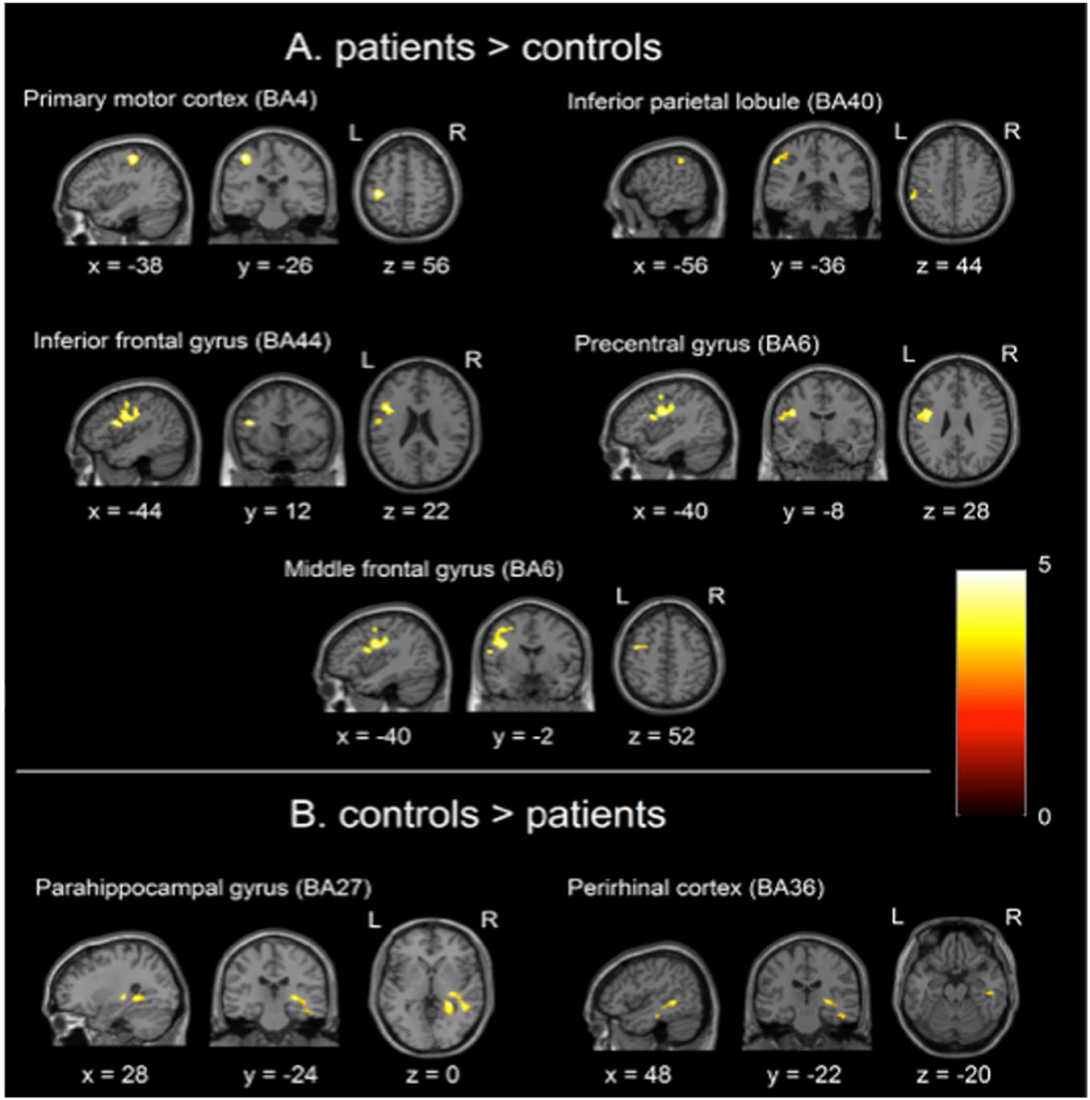
Neuronal activation for the cluster level analysis (FWE, *P* < 0.05) where activation is greater in (**A**) patients versus controls and (**B**) controls versus patients, for the response to all movement >rest. MNI coordinates are given (x, y, z mm) for the most significant voxel in the cluster. L = left hemisphere, R = right hemisphere. Colour (including colour bars) corresponds to *T*-scores.

No significant differences in cortical activation were found between the two types of movement (forward flexion and abduction) when tested across all participants, and no effect was found for the interaction movement type*patient group (FWE, *P* < 0.05). Consequently all movement blocks were considered together when comparing control and patient groups. The cluster analysis (FWE, *P* < 0.05) yielded five areas where activation was greater in patients compared to controls for the contrast all movement >rest (Table 1A, Fig. 2). All clusters were located in the left hemisphere and included primary motor cortex (BA4) and supramarginal gyrus (BA40). Though the most significant voxel in the cluster did reside within the primary motor cortex, it should be noted that the cluster also encompassed part of the somatosensory cortex (BA3), as can be seen in Fig. 2. The remaining three clusters were in the frontal lobe, specifically in inferior frontal gyrus (BA44), precentral gyrus (BA6) and middle frontal gyrus (BA6), of which the latter two regions are considered part of the premotor cortex.

**Table 1:** Brain regions from the cluster level correction (FWE, *P* < 0.05) showing significant differences in activation for all movement >rest for the following contrasts: (A) patient greater than controls, and (B) controls greater than patients.

Region	BA	Cluster size	T-score	MNI coordinates		
				x	y	z
**A. Contrast: patients > controls**						
Primary motor cortex	4	430	5.22	−38	−26	56
Supramarginal gyrus	40	430	4.24	−56	−36	44
Inferior frontal gyrus	44	769	4.87	−44	12	22
Precentral gyrus	6	769	4.54	−40	−8	28
Middle frontal gyrus	6	769	4.22	−40	−2	52
**B. Contrast: controls >patients**						
Parahippocampal gyrus	27	719	4.93	28	−24	0
Perirhinal cortex	36	719	3.73	48	−22	−20

MNI coordinates of the most significant voxel (x, y, z mm) in the cluster are given, along with the corresponding brain region for this voxel and the closest Brodmann Area (BA) corresponding to that region.

Results from the voxel-level correction (FWE, *P* < 0.05), using these five clusters as ROIs, identified a single voxel where activation was greater in patients compared to controls within the primary motor cortex (BA4), at the cluster (MNI) coordinates x = −38, y = −26 z = 56. The presence of this voxel was tested for in each patient separately using the contrast movement >rest. Activation at this voxel coordinate was present in all patients except the single patient with clinical scores on WOSI and OSIS resembling a normal shoulder; nor was the voxel present in any of the control subjects.

The contrast greater activation in controls compared to patients, using a cluster level correction (FWE, *P* < 0.05) yielded activation in the right hemisphere in a region spanning parahippocampal gyrus (BA27) and perirhinal cortex (BA36), with peak threshold at the (MNI) coordinates (x = 28, y = −24, z = 0) (see Table 1B, Fig. 2B).

## Discussion

There has been much work looking at compensatory activation in stroke patients^70^, in shoulder movement^71^, preclinical Parkinson’s disease^72^ and Huntington’s disease^73^. Further, there has been some therapeutic translation, where constraint-induced movement therapy has been deployed to attempt motor cortex reorganisation^74–76^. fMRI has never previously been applied to shoulder instability, although cortical activation abnormalities have been explored in shoulder apprehension^40^.

The patients showed different cortical activation compared to the controls. At a cluster level there were five areas of greater activation in patients compared to controls, located in the left hemisphere and consisting of primary motor cortex (BA4) and supramarginal gyrus (BA40), inferior frontal gyrus (BA44), precentral gyrus (BA6) and middle frontal gyrus (BA6). At a voxel level, a single voxel was found within primary motor cortex (BA4) at the coordinates -38 -26 56. Retrospective analysis revealed that activation of the coordinate was present in all the patients with abnormal WOSI and OSIS scores, but not the patient with normal scores. This cortical region is associated with complex motor tasks that involve a high degree of co-ordination^77^.

The voxel (-38 -26 56) is located within BA4 in the left hemisphere. Grefkes *et al*.^42^ have shown this location to have an inhibitory effect at distant sites within the motor cortex, particularly evident in chronic stroke patients, where the location was found to contribute to impaired motor function on the affected side. Such chronicity is also characteristic of our patient group, and suggests that a centrally-driven inhibition might lead to global shoulder instability, with further cortical activation occurring during the unstable movement. It is well known that physiotherapy induces motor cortex re-organisation and reduced activation^78^, perhaps representing a return to a normal motor cortex. This would be consistent with our finding in the treated patient with a normal scores.

When testing for greater cortical activity in patients compared to controls, the most significant voxel for a cluster of neural activity was found in primary motor cortex (BA4), which as noted also encompassed somatosensory cortex (BA3). There is a tight link between sensory processing and movement production^79^. Articular mechanoreceptors are postulated to serve a role in sensorimotor control over functional joint stability^80^. Proprioception-related brain activation also highlights a key role for the supramarginal gyrus^81–83^, part of the somatosensory association cortex, which interprets tactile sensory data and is involved in the perception of space and limb location^84,85^. Ben-Shabat *et al*.^21^ found supramarginal gyrus and dorsal premotor cortex to be associated with proprioception in healthy participants; in stroke-affected participants the main difference in proprioception-related brain activation was reduced laterality in the right supramarginal gyrus, which the authors suggest may be associated with decreased proprioception^21^. In previous studies early learning of sequential motor tasks has been associated with an increase in task-evoked BOLD response within premotor cortex, supplementary motor areas and parietal regions^86–88^. Bassett *et al*.^89^ showed that learning a simple motor skill induced an autonomy of sensorimotor systems consistent with a neural efficiency hypothesis: cortical systems tend to economize resources as learning progresses. In the current study participants performed a relatively simple, though atypical, motor sequence. That patients showed increased activity compared to controls in premotor cortex (i.e. precentral gyrus [BA6] and middle frontal gyrus [BA6]), sensorimotor systems and parietal regions (specifically supramarginal gyrus) is consistent with the notion that they are working harder to achieve motor stability in a task of low cortical demand, not involving high level co-ordination.

Compared to the patient group, controls presented greater cortical activity in the parahippocampal gyrus (BA27) and perirhinal cortex (BA36). Collectively these two regions are major sources of polysensory input to the amygdala^90^. Tract-tracing studies in rodents and monkeys suggest that these regions differ in their anatomical connectivity with sensory and association areas and with hippocampal subfields^91,92^. In rats the strongest input to area 36 of the perirhinal cortex arises from anterior and ventral temporal association areas known to receive strong projections from somatosensory and auditory areas^91^. The afferent inputs to rat perirhinal areas 35 and 36 are dominated by sensory inputs from the olfactory, somatosensory and auditory as well as visual modalities^93^. Using resting-state fMRI Libby *et al*.^94^ demonstrated preferential perirhinal cortex connectivity with an anterior temporal and frontal cortical network and preferential parahippocampal cortex connectivity with a posterior medial temporal, parietal and occipital network. However, because anatomical tracer studies are not feasible in humans, it is not known whether perirhinal and parahippocampal cortex exhibit differential structural connectivity with higher neocortical regions, hippocampal subfields, or what is their precise connectivity with somatosensory cortex. One hypothesis for this increased activation in controls within perirhinal and parahippocampal cortex is that the processed information from the somatosensory regions required to maintain shoulder stability during the movement task is being fed forward to the perirhinal and parahippocampal cortex, then distributed for further processing. However, further research will be required to fully understand the functional and anatomical connectivity of the somatosensory cortex with the perirhinal cortex and parahippocampal cortex.

### Limitations and future directions

Patients with complex shoulder instability suffer from a condition that is difficult to categorise and is part of a continuum disorder, and so variation is inevitable in any patient group. Clearly large-scale multicentre studies are required to represent the full spectrum of disability within the group.

Patients with complex shoulder instability develop their instability spontaneously without any known precipitating trauma or event, although some of our patients had suffered a serious psychological traumatic episode. The potential role of psychology deserves future exploration. Although little attention has been paid to it, the psychological component of shoulder instability was recognised in the 1970s by Rowe *et al*.^95^, who found that a subset of patients suffered from conditions ranging from simple depression to post-traumatic stress disorder related to sexual assault. Further, these patients were resistant to treatment^96^.

Our retrospective analysis of the patient who had essentially recovered with a normal WOSI score, found cortical activation similar to the control group. This would suggest that the instability at a cortical level is plastic, although it is unclear whether it is causal in the instability or merely reflects the change in movement of the shoulder. Longitudinal studies that monitor patients through their treatment could shed light on the mechanisms involved in this rehabilitation.

## Conclusion

We have for the first time demonstrated a difference in brain activity during a shoulder movement sequence between patients with complex shoulder instability and healthy controls. Specifically, increased brain activity was found within the primary motor cortex (a cluster which stretched between BA4 and BA3), supramarginal gyrus (BA40), inferior frontal gyrus (BA44), premotor cortex (i.e. precentral gyrus (BA6) and middle frontal gyrus (BA6)) in patients compared to controls. These findings are consistent with the notion that patients are in some sense working harder or differently to maintain shoulder stability, exhibiting neural activity akin to early learning of a motor sequence. Further, that instability is likely to be centrally driven rather than in response to peripheral damage, although the mechanism is unknown. Controls, compared to patients, demonstrate increased neural activity in the perirhinal and parahippocampal cortex. The exact role of these two regions is unclear, but may relate to the processing and onward transmission of somatosensory information required to maintain shoulder stability.

Clinically this group of patients is often poorly treated, as healthcare professionals are unable to explain the recurrent dislocations and instability. This is the first demonstration of an objective difference in brain activation. This may help to dispel the myth that these patients are inducing their instability and dislocations as a form of attention-seeking behaviour, and potentially offers opportunities for physiotherapy interventions.
